# Effects of realgar-indigo naturalis formula on a zebrafish tumor xenograft model induced by human acute promyelocytic leukemia cells: antitumor activity, hepatotoxicity, and transcriptomic analysis

**DOI:** 10.3389/fphar.2025.1619352

**Published:** 2025-07-18

**Authors:** Donghan Bai, Zihao Zhang, Jingyue Gao, Qiaochu Wang, Remy Macdonald, Ziwen Xu, Shumin Chen, Nanxi Huang, Lu Luo

**Affiliations:** ^1^ Institute of Chinese Materia Medica, China Academy of Chinese Medical Sciences, Beijing, China; ^2^ Department of Biochemistry and Molecular and Cellular Biology, Georgetown University, Washington, DC, United States; ^3^ Teachers College, Columbia University, New York, NY, United States; ^4^ Department of Statistics, George Mason University, Fairfax, VA, United States; ^5^ Department of Nursing, The University of Melbourne, Melbourne, VIC, Australia; ^6^ Institute of Acupuncture and Moxibustion, China Academy of Chinese Medical Sciences, Beijing, China; ^7^ Pharmacological and Pharmaceutical Sciences, College of Pharmacy, University of Houston, Houston, TX, United States

**Keywords:** leukemia, Realgar-Indigo naturalis formula (RIF), all-trans retinoic acid, zebrafish, transcriptomic analysis

## Abstract

**Background:**

Leukemia is a malignant hematologic disease that poses a serious threat to human health. Realgar-Indigo Naturalis formula (RIF), a traditional Chinese medicine formula, have demonstrated anti-tumor activity, but its mechanisms of action remain unclear.

**Objective:**

This study aimed to establish a zebrafish HL-60 tumor transplantation model to investigate the anti-leukemic effects of RIF and explore its underlying mechanisms.

**Methods:**

The zebrafish HL-60 tumor transplantation model was established, with RIF as the intervention drug and all-trans retinoic acid (ATRA) as the control. Anti-tumor efficacy was assessed via pharmacodynamic analysis. Transcriptomic analysis further elucidated gene expression profiles, differentially expressed genes, and relevant biological pathways.

**Results:**

RIF significantly reduced tumor cell fluorescence intensity, demonstrating anti-tumor efficacy. Additionally, it improved HL-60-induced liver damage in zebrafish, as evidenced by a reduction in fatty vacuolar degeneration in liver tissue. Transcriptomic analysis revealed that ATRA mainly affected the FoxO signaling pathway, PI3K-Akt signaling pathway, apoptosis, and complement and coagulation cascades in leukemia treatment. RIF primarily influenced the ubiquitin-proteasome system, ferroptosis, and glutathione metabolism. The combination of ATRA and RIF also affected autophagosome and lysosome pathways, in addition to the aforementioned pathways.

**Conclusion:**

RIF exhibit significant anti-tumor effects through modulation of multiple pathways. This study provides a theoretical foundation for the potential clinical application of RIF in leukemia treatment.

## 1 Introduction

Leukemia is a malignant hematologic disease that poses a serious threat to human health, significantly impacting patients’ quality of life and survival expectancy. Global epidemiological data indicate a continuous rise in the annual incidence of leukemia, with a substantial number of new cases reported each year. The pathogenesis of leukemia is highly complex, involving the intricate interplay of genetic and environmental factors, which disrupt the normal proliferation and differentiation of hematopoietic stem cells (Wachter and Pikman, 2024). This abnormality suppresses normal hematopoiesis, leading to severe clinical manifestations such as anemia, hemorrhage, and immune dysfunction (Whiteley et al., 2021; Kantarjian et al., 2024). While global epidemiological data indicate a continuous rise in the annual incidence of leukemia, the situation in China presents a specific challenge. According to a 2024 analysis of national cancer data, leukemia ranked as the 10th most frequently diagnosed cancer in both Chinese men and women in 2022. More critically, it stands as a leading cause of cancer-related burden (Disability-Adjusted Life Years) among children and adolescents under 20, underscoring its profound impact on younger populations (Diao et al., 2025).

Currently, leukemia treatment strategies primarily include chemotherapy, radiotherapy, hematopoietic stem cell transplantation, and targeted therapy (Kantarjian et al., 2024). Although chemotherapy can effectively slow disease progression to some extent, its nonspecific cytotoxicity results in significant damage to normal cells while eliminating leukemia cells. This collateral damage often leads to severe adverse effects, including myelosuppression, gastrointestinal reactions, and hepatic and renal dysfunction, thereby considerably affecting patients’ quality of life and treatment adherence (Karunarathna et al., 2024). In order to avoid the adverse effects of chemotherapy, non - chemotherapeutic strategies have been constantly explored for the treatment of APL (Mi et al., 2012; Mathews et al., 2010; Jain et al., 2013).

Realgar-Indigo Naturalis formula (RIF), a traditional Chinese medicine (TCM) formulation specifically designed for the treatment of newly diagnosed acute promyelocytic leukemia (APL), have demonstrated significant therapeutic advantages and are widely used in clinical practice (Huang et al., 2022; Xiang et al., 2009; Xiang et al., 2010; Bai et al., 2025). The primary components of this formulation include *Indigo Naturalis, Realgar, Pseudostellaria heterophylla, and Salvia miltiorrhiza* (Zhang et al., 1981). Among these, Indirubin derivatives, a key bioactive compound in Indigo Naturalis, effectively inhibits tumor cell nucleic acid metabolism, thereby suppressing leukemia cell proliferation (Nam et al., 2012). The arsenic sulfide in Realgar exhibits direct antitumor activity by inhibiting leukemia cell growth (Zhang et al., 2009; Zhu et al., 2001). Pseudostellaria heterophylla enhances immune function by strengthening the spleen, replenishing Qi, and nourishing blood, while Salvia miltiorrhiza exerts anticoagulant and anti-inflammatory effects, improving the tumor microenvironment and regulating blood circulation (Gui et al., 2025; You et al., 2021; Guo et al., 2014; Jung et al., 2020). The synergistic actions of these components contribute to the overall therapeutic effects of RIF, including clearing heat and detoxifying, invigorating Qi, and nourishing blood, making it a promising treatment option for leukemia. Compared with conventional chemotherapeutic agents, RIF are associated with fewer adverse effects and are better tolerated by patients. For instance, a clinical study on newly diagnosed APL patients demonstrated that those treated with RIF had a significantly higher 5-year disease-free survival rate than the control group, highlighting its potential in improving long-term prognosis (Liu et al., 2015). However, despite its remarkable clinical efficacy, the underlying mechanisms of RIF remain inadequately understood. Key aspects such as drug metabolism, molecular targets, and potential interactions with other therapeutic agents require further investigation to fully elucidate its pharmacological properties and optimize its clinical application.

As an emerging experimental model organism, zebrafish offer unique advantages in cancer research. The zebrafish genome shares approximately 87% similarity with the human genome, exhibiting significant conservation in key biological processes such as the cell cycle, tumor suppressor genes, and oncogenes (Dooley and Zon, 2000). This high degree of genetic homology enables zebrafish models to effectively recapitulate the initiation and progression of human tumors, making them a reliable platform for studying tumor pathogenesis and evaluating therapeutic agents (Liu and Leach, 2011; Feitsma and Cuppen, 2008). Compared with traditional mouse tumor models, zebrafish xenograft models provide several advantages, particularly during the larval stage, when the immune system is not fully developed. This immunodeficient state eliminates the need for immunosuppressive agents, simplifying experimental procedures and reducing potential confounding effects, thereby enhancing the efficiency and accuracy of tumor studies (Yan et al., 2019).

In this study, we established a zebrafish tumor xenograft model using human acute promyelocytic leukemia (APL) cells (HL-60) and evaluated the therapeutic effects of RIF, with all-trans retinoic acid (ATRA) serving as a control treatment. By integrating pharmacodynamic assessments and transcriptomic analysis, we investigated the impact of RIF on gene expression profiles in zebrafish. Differentially expressed genes (DEGs) were identified, and bioinformatics analysis was performed to elucidate the biological processes and signaling pathways involved. At the molecular level, our findings provide insights into the antitumor mechanisms of RIF, offering a theoretical basis for further clinical validation and potential therapeutic applications.

## 2 Materials and methods

### 2.1 Experimental animals and cell culture

Zebrafish were maintained in a dedicated aquatic system at 28°C, with water quality parameters set as follows: 200 mg of instant ocean salt per liter of reverse osmosis water, conductivity of 450–550 μS/cm, pH ranging from 6.5 to 8.5, and water hardness of 50–100 mg/L CaCO_3_. The use of experimental animals was approved under license number SYXK (Zhejiang) 2022–0,004, and husbandry conditions complied with the standards of the Association for Assessment and Accreditation of Laboratory Animal Care International (AAALAC) (certification number: 001,458). Ethical approval for the study was granted under protocol number IACUC-2024–10,275–01. Human acute promyelocytic leukemia (APL) cells (HL-60) were cultured in RPMI-1640 medium supplemented with 10% fetal bovine serum (FBS) and 1% penicillin-streptomycin (P/S). Cells were maintained at 37°C in a humidified incubator with 5% CO_2_ to ensure optimal growth conditions.

### 2.2 Reagents

DMSO (20,240,424, China National Pharmaceutical Group Corp., China). All-trans retinoic acid (ATRA, 156,718, MedChemExpress, China). Methyl cellulose (C2004046, Aladdin Biochemical Technology Co., Ltd., China). Vybrant™ CM-Dil Cell-Labeling Solution (CM-Dil, 2,776,007, Thermo Fisher Scientific (China) Co., Ltd., United Sates). Phosphate-buffered saline (PBS, BL601A, Biosharp, China). Penicillin-Streptomycin (P/S, 2,585,616, Gibco, China). RPMI-1640 Medium (2,842,153, Thermo Fisher Scientific, China). Fetal bovine serum (FBS, N2862027P, Gibco, China). Anhydrous ethanol (20,240,426, China National Pharmaceutical Group Corp., China). 4% Tissue Fixative (240,004,005, Solarbio, China). Xylene (20,240,704, China National Pharmaceutical Group Corp., China). Eosin staining solution (20,220,120, Shanghai Yihe Biotechnology Co., Ltd., China). Mayer’s Hematoxylin Staining Solution (20,220,120, Shanghai Yihe Biotechnology Co., Ltd., China). Neutral resin (330A021, Solarbio, China). High-quality sectioning paraffin (melting point 54°C–56°C, 20,201,020, Shanghai Huayong Paraffin Co., Ltd., China). High-quality sectioning paraffin (melting point 62°C–64°C, 20,210,828, Shanghai Huayong Paraffin Co., Ltd., China). DEPC Water (AM9922, Ambion, United Sates). Oligo (dT) Magnetic Bead mRNA Enrichment (Optimal Dual-mode mRNA Library Prep Kit, LR00R96, BGI, China).

### 2.3 Determination of maximum tolerated concentration (MTC)

Wild-type AB strain zebrafish, aged 3 days post-fertilization (3 dpf), were randomly selected and placed into a 6-well plate for treatment. Each well contained 30 zebrafish (experimental group). The samples were administered to the experimental group as aqueous solutions (concentrations of the samples are detailed in Tables 1,2). A normal control group was also established, with a water volume of 3 mL per well. After 2 days of continuous treatment at 35°C, the maximum tolerated concentration (MTC) of the sample was evaluated based on the zebrafish’s response.

**TABLE 1:** Results of the experiment on exploring the anti-tumor efficacy concentrations of samples (n = 30).

Group	Concentration (μg/mL)	Number of deaths (tails)	Mortality rate (%)	Phenotype VS control
Control	-	0	0	-
ATRA	0.195	0	0	Similar
	0.391	0	0	Worse
	0.781	0	0	Worse
	1.56	27	90	-
	3.12	30	100	-
RIF	125	0	0	Similar
	250	0	0	Similar
	500	1	3	Worse
	1,000	3	10	Worse
	2000	9	30	-

**TABLE 2:** Results of the experiment on exploring the anti - tumor efficacy concentrations of samples (n = 30).

Group	Concentration of RIF(μg/mL)	Concentration of ATRA (μg/mL)	Number of deaths (tails)	Mortality rate (%)	Phenotype VS control
Control	-		0	0	-
RIF + ATRA	15.6	12.2	0	0	Similar
	31.2	24.4	0	0	Similar
	62.5	48.8	0	0	Similar
	125	97.7	0	0	Similar
	250	195	1	3	worse

### 2.4 Evaluation of antitumor growth efficacy

Human acute promyelocytic leukemia (HL-60) cells were labeled with CM-DiI fluorescence dye and transplanted into the yolk sac of 2-day post-fertilization (dpf) wild-type AB strain zebrafish via microinjection. Approximately 200 cells were transplanted into each zebrafish to establish a zebrafish tumor xenograft model. This cell number was chosen based on established protocols for HL-60 cell xenografts in zebrafish and our preliminary optimization experiments, which indicated that this number provides consistent tumor engraftment and growth detectable by fluorescence microscopy within the experimental timeframe, without causing undue acute toxicity to the zebrafish larvae. The transplanted zebrafish were maintained at 35°C until they reached 3 dpf. At 3 dpf, zebrafish with consistent tumor cell transplantation were selected under a microscope and randomly distributed into 6-well plates, with 30 zebrafish per well (experimental group). The samples were administered to the experimental group as aqueous solutions (sample concentrations are detailed in Table 3), and a model control group was established. Each well contained 3 mL of water. After 2 days of continuous treatment at 35°C, 10 zebrafish from each experimental group were randomly selected. Fluorescence microscopy images were captured, and data were collected using NIS-Elements D 3.20 image processing software. The antitumor effect of the samples was evaluated by analyzing the fluorescence intensity of the tumor cells, which reflects the inhibition of tumor (HL-60) growth. Statistical results were presented as mean ± standard error (mean ± SE), and data were analyzed using SPSS 26.0 software. A p-value of <0.05 was considered statistically significant.

**TABLE 3:** Results of the anti - tumor (HL - 60) efficacy evaluation experiment of samples (n = 10).

Group	Fluorescence intensity of tumor cells (pixels, mean ± SE)
Model	1,215,441 ± 73,366
ATRA	827,610 ± 141,430*
RIF	863,415 ± 126,211*
RIF + ATRA	748,717 ± 76,118***

Compared with the model control group, *p < 0.05, ***p < 0.001.

### 2.5 Liver histopathological sections

**FIGURE 1:**
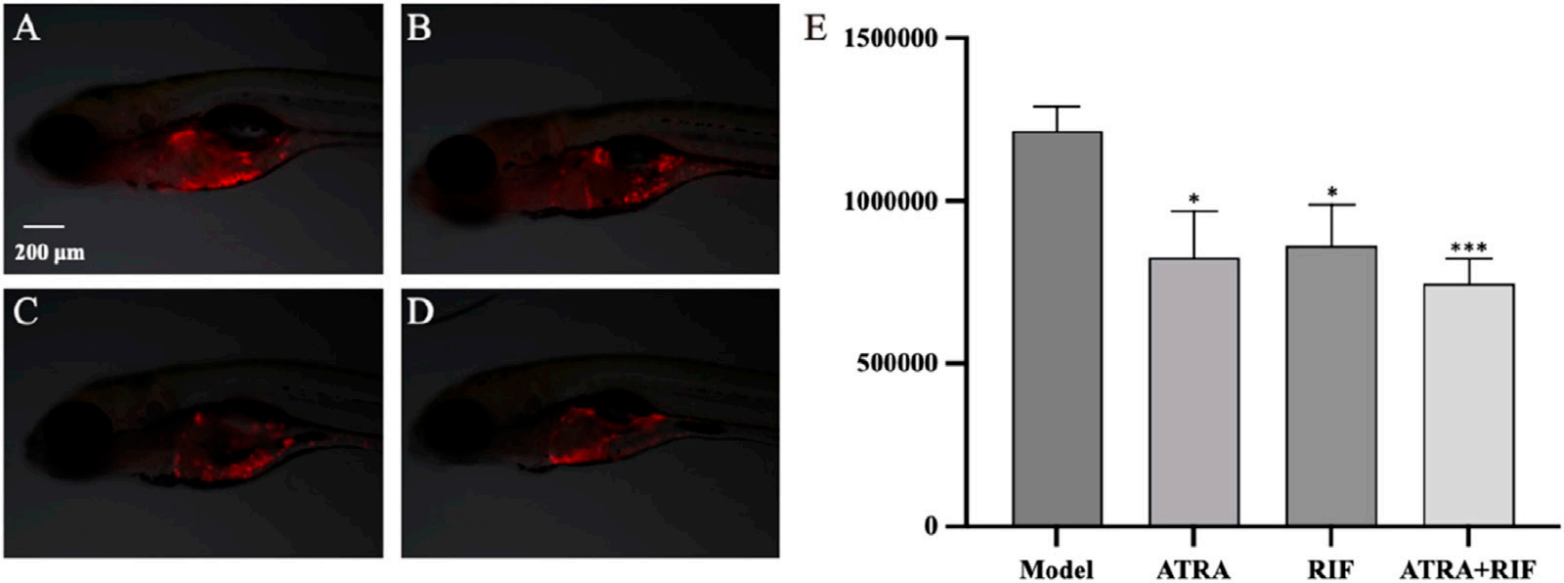
Growth of zebrafish tumor cells (HL - 60) after sample treatment **(A)** Model control group; **(B)** ATRA treatment group; **(C)** RIF treatment group; **(D)** ATRA + RIF combined treatment group. Compared with the model control group, the fluorescence intensity of tumor cells in the ATRA group and the RIF group decreased significantly (*p < 0.05), and that in the ATRA + RIF group decreased significantly (***p < 0.001). **(E)** Statistical analysis chart of the fluorescence intensity of tumor cells (pixels), and the data are expressed as mean ± SE.

**FIGURE 2:**
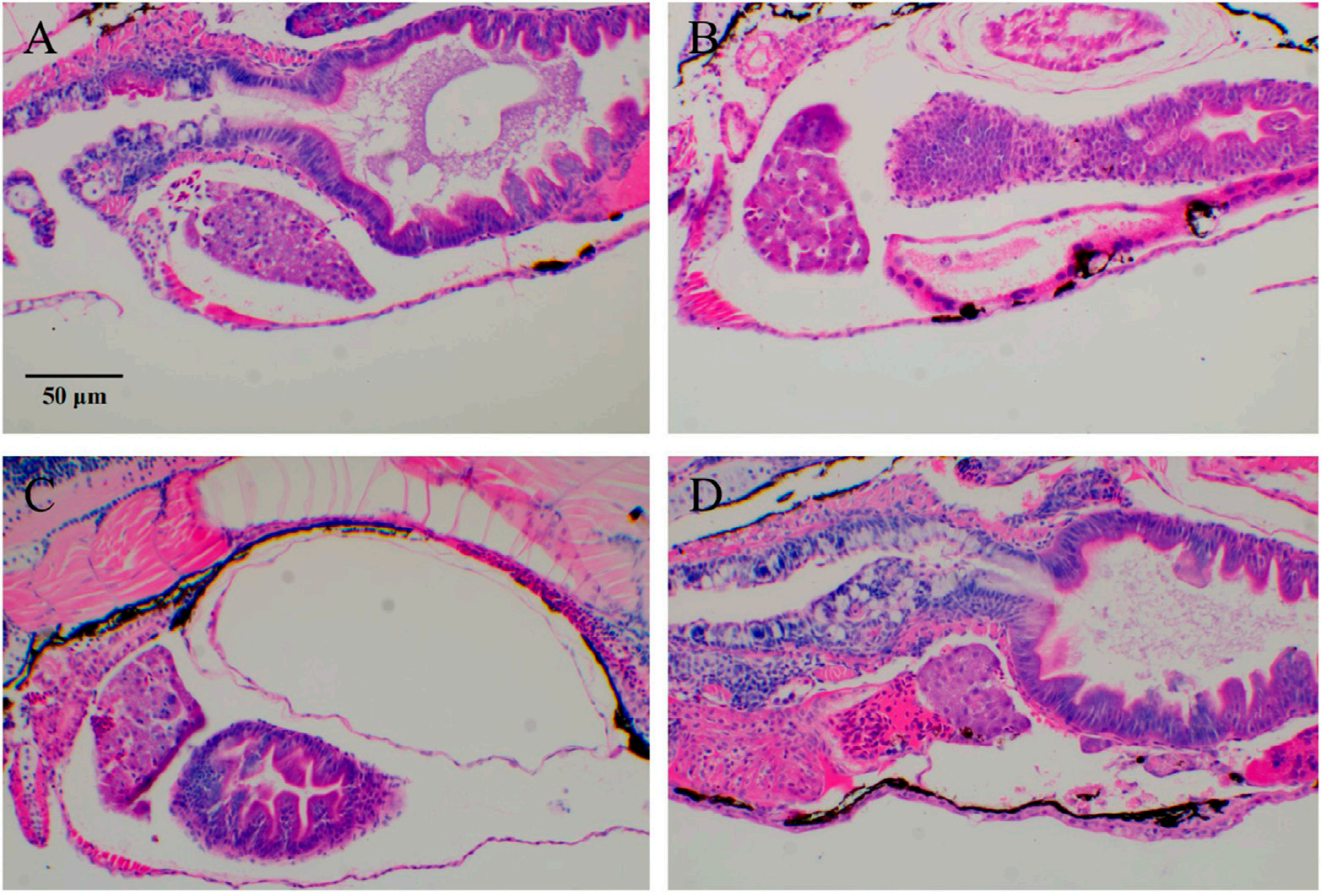
Typical images of zebrafish tumor cell (HL-60) migration after RIF intervention. **(A)** Model group; **(B–D)** ATRA, RIF, and ATRA + RIF. In the liver tissue of zebrafish from the model control group, extensive fat vacuolar degeneration (indicated by yellow arrows) is observed, confirming the successful establishment of the model. In contrast, the liver tissue of zebrafish in the treatment groups shows a significant reduction in fat vacuolar degeneration, suggesting that ATRA and Compound Huangdai Tablets have a potential protective effect against liver injury.

**FIGURE 3:**
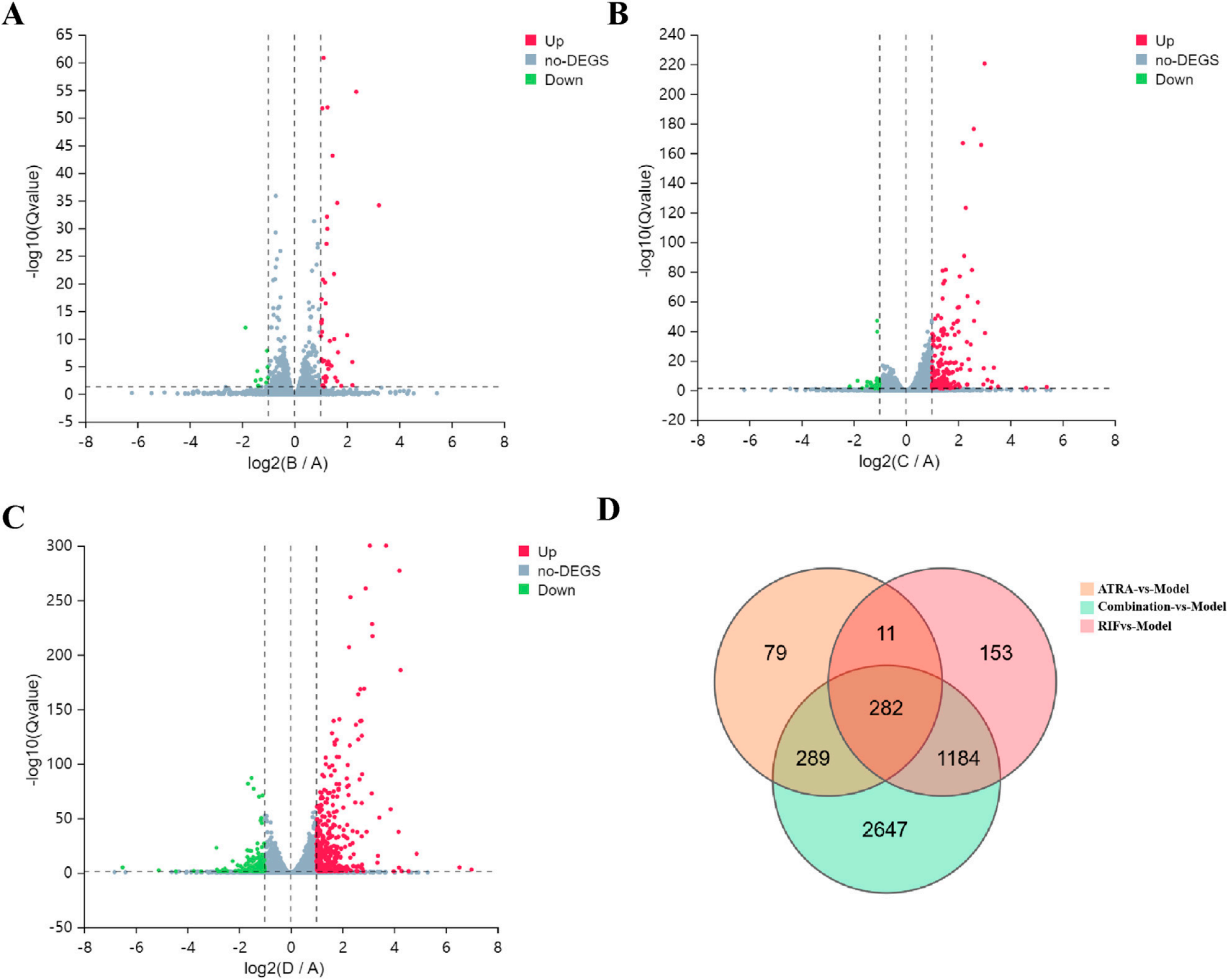
Transcriptomic profiling of differentially expressed genes (DEGs) across treatment groups. **(A–C)** Volcano plots show DEGs under |log2FC| ≥ 1 and Q-value ≤0.05 thresholds for **(A)** ATRA-vs-Model (45 upregulated, red; 10 downregulated, green), **(B)** RIF-vs-Model (168 upregulated, red; 29 downregulated, green), and **(C)** Combination-vs-Model (385 upregulated, red; 189 downregulated, green). Dashed lines demarcate significance thresholds. **(D)** Venn diagram illustrates overlapping DEGs (n = 282) common to all three comparisons, indicating shared transcriptional perturbations.

HL-60 cells were labeled with CM-DiI fluorescence dye and transplanted into the yolk sac of 2-day post-fertilization (dpf) wild-type AB strain zebrafish via microinjection. Approximately 200 cells were transplanted into each zebrafish to establish the zebrafish tumor xenograft model. The transplanted zebrafish were maintained at 35°C until they reached 3 dpf. At 3 dpf, zebrafish with consistent tumor cell transplantation were selected under a microscope and randomly distributed into 6-well plates, with 30 zebrafish per well (experimental group). The samples were administered to the experimental group as aqueous solutions (sample concentrations are shown in Figures 1–3), and a model control group was set up, with 3 mL of water in each well. After 2 days of continuous treatment at 35°C, zebrafish from each group were subjected to fixation, dehydration, embedding, sectioning, and hematoxylin and eosin (HE) staining. The liver tissues were analyzed histopathologically to evaluate the protective effect of the sample on tumor-induced liver damage.

### 2.6 Transcriptomic study

HL-60 cells were transplanted into the yolk sac of 2-day post-fertilization (dpf) wild-type AB strain zebrafish via microinjection. Approximately 200 cells were transplanted into each zebrafish to establish the zebrafish tumor xenograft model. The transplanted zebrafish were maintained at 35°C until they reached 3 dpf. At 3 dpf, zebrafish with consistent tumor cell transplantation were selected under a microscope and randomly distributed into 6-well plates, with 30 zebrafish per well (experimental group). The experimental groups were treated with the following concentrations: ATRA 97.7 ng/mL, Compound Huangdai Tablets 125 μg/mL, and Compound Huangdai Tablets 125 μg/mL + ATRA 97.7 ng/mL. A model control group was also set up, with 3 mL of water per well. Each group had 3 biological replicates. After 2 days of treatment at 35°C, the zebrafish were thoroughly washed with ultrapure water. They were then transferred into 1.5 mL microcentrifuge tubes (30 zebrafish per tube). After the liquid was removed, the samples were immediately placed in liquid nitrogen for 3 min, followed by storage at −80°C for subsequent transcriptomic analysis.

Following sample collection (30 zebrafish per tube, 3 biological replicates per group, as described above), total RNA was extracted from the pooled zebrafish larvae using TRIzol reagent (Invitrogen, Cat# 15596026). The quality and quantity of the extracted total RNA were assessed. Samples meeting quality control standards underwent DNase I digestion. mRNA was then enriched from the total RNA using Oligo (dT) magnetic beads (Optimal Dual-mode mRNA Library Prep Kit, BGI, Cat# LR00R96). The enriched mRNA was fragmented, and first-strand cDNA was synthesized using random primers. Second-strand cDNA was synthesized using dUTP instead of dTTP. The resulting cDNA underwent end-repair, ‘A’-tailing, and adapter ligation. The ligated products were then subjected to PCR amplification. UDG enzyme was used to digest the U-labeled second strand template before PCR amplification. PCR products were purified and their quality was assessed.

The PCR products were denatured into single strands and circularized to form single-strand circular DNA libraries. Uncircularized linear DNA molecules were digested. Sequencing was performed on the DNBSEQ platform (MGI 2000, MGI, China) after DNA nanoball (DNB) formation through rolling circle replication. Raw sequencing reads were filtered to remove low-quality reads, adapter contaminants, and reads with high N content. Quality control metrics (Q20, Q30) were assessed (see Tables 1, 2 in results for quality statistics). The clean reads were then aligned to the zebrafish reference genome using HISAT (Hierarchical Indexing for Spliced Alignment of Transcripts). Gene expression levels were quantified as read counts. Differential gene expression analysis between groups was performed using standard bioinformatics pipelines. Genes with a |log2FoldChange| ≥ 1 and an adjusted p-value (Q-value) ≤0.05 were considered differentially expressed genes (DEGs).

Functional enrichment analysis of the differentially expressed genes (DEGs) was performed using the standard pipeline provided by BGI (Shenzhen, China). Specifically, Gene Ontology (GO) enrichment analysis was conducted to categorize genes based on biological process, cellular component, and molecular function. Kyoto Encyclopedia of Genes and Genomes (KEGG) pathway enrichment analysis was performed to identify significantly enriched metabolic or signaling pathways. These analyses utilize hypergeometric tests for enrichment and correct for multiple testing.

### 2.7 Statistical Analysis

All data were presented in the form of mean ± standard error of the mean (SEM). To determine the significance of differences, one - way analysis of variance was employed. P-value less than 0.05 was regarded as indicating a significant difference between the groups.

## 3 Result

### 3.1 MTC determination

Under the conditions of this experiment, the maximum tolerated concentration (MTC) of all - trans retinoic acid (ATRA) for anti - tumor efficacy was 0.195 μg/mL, the MTC of Compound Realgar Natural Indigo Tablet for anti - tumor efficacy was 250 μg/mL, and the MTC of the combined application of Compound Realgar Natural Indigo Tablet and ATRA for anti - tumor efficacy was Compound Realgar Natural Indigo Tablet 125 μg/mL + ATRA 97.7 ng/mL. Based on these MTC results, where zebrafish survival and phenotype were assessed (Tables 1, 2), the concentrations selected for subsequent anti-tumor efficacy and transcriptomic studies were those that were well-tolerated (≤MTC, with minimal mortality and normal phenotype) and represented potentially effective doses: RIF 125 μg/mL, ATRA 97.7 ng/mL, and the combination of RIF 125 μg/mL + ATRA 97.7 ng/mL.

### 3.2 RIF inhibits HL-60 induced tumor growth

Under the experimental conditions, ATRA at a concentration of 97.7 ng/mL, Compound Realgar Natural Indigo Tablet at a concentration of 125 μg/mL, and their combination (Compound Realgar Natural Indigo Tablet 125 μg/mL + ATRA 97.7 ng/mL) all demonstrated significant anti - tumor efficacy. The specific experimental data and relevant results are presented in Table 3 and Figure 1.

### 3.3 The improvement effect of RIF on HL-60-induced liver injury in zebrafish

Most chemotherapy drugs, while effective in killing leukemia cells, also cause significant damage to normal cells, leading to liver and kidney dysfunction and severely affecting the patient’s quality of life and treatment adherence. To identify more effective and safer treatment options, and to evaluate the protective effects of ATRA and Compound Huangdai Tablets on HL-60-induced zebrafish liver injury, we established a zebrafish liver injury model in this experiment and observed the effects of different treatment groups. Under the experimental conditions, the liver tissue of the model control group zebrafish showed extensive fat vacuolar degeneration (indicated by yellow arrows), confirming the successful establishment of the model. In contrast, the zebrafish liver tissue in the sample treatment groups exhibited a marked reduction in fat vacuolar degeneration, suggesting that ATRA and Compound Huangdai Tablets have a certain degree of improvement in liver injury. Detailed results are shown in Figure 2.

### 3.4 Transcriptomic analysis

#### 3.4.1 Differential gene expression profiling

Transcriptomic profiling under |log2FC| ≥ 1 (indicating at least a two-fold change) and Qvalue ≤0.05 (false discovery rate corrected p-value) thresholds revealed a treatment-dependent modulation of gene expression profiles across experimental groups. The |log2FC| ≥ 1 cutoff was chosen as it is a commonly accepted standard in transcriptomic analyses to identify genes with biologically meaningful changes in expression, ensuring a balance between sensitivity and specificity for downstream functional analysis. Specifically, the ATRA-vs-Model comparison yielded 45 upregulated and 10 downregulated genes, while RIF-vs-Model showed 168 upregulated and 29 downregulated genes. Notably, the Combination-vs-Model group exhibited the most pronounced transcriptional changes, with 385 upregulated and 189 downregulated genes. A core set of 282 overlapping DEGs was identified across all comparisons, suggesting shared regulatory pathways impacted by individual and combinatorial treatments. Volcano plots (Figures 3A–C) visually resolved the differential expression patterns in each comparison, while Venn analysis (Figure 3D) systematically mapped the intersection of DEGs, highlighting conserved molecular targets. This integrated approach underscores the combinatorial treatment’s amplified transcriptional impact and identifies critical overlapping genes for mechanistic interrogation.

#### 3.4.2 Multi-pathway regulatory mechanisms of ATRA in treating APL

**FIGURE 4:**
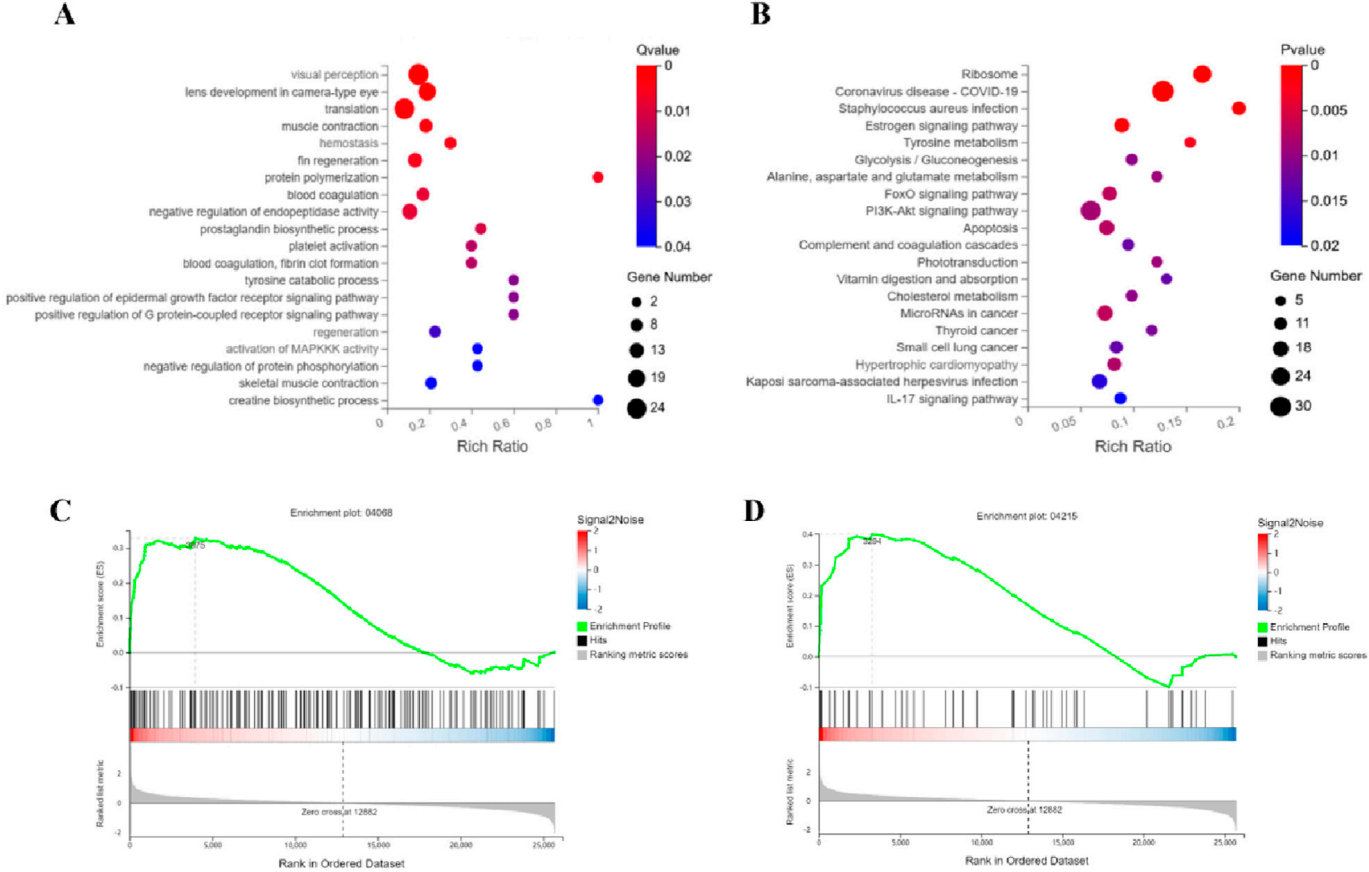
Integrated mechanisms of ATRA in APL therapy. Functional analyses identify key pathways mediating ATRA’s therapeutic effects: **(A)** GO analysis links ATRA to muscle contraction, creatine synthesis, and coagulation; **(B)** KEGG analysis highlights altered FoxO and PI3K-Akt signaling alongside apoptotic regulation; Gene Set Enrichment Analysis (GSEA) further validates FoxO **(C)** and apoptosis **(D)** as critical regulatory axes.

Integrated GO and KEGG analyses reveal that ATRA significantly modulates leukemia-associated biological pathways. In the GO enrichment analysis of the ATRA-vs-Model group (Figure 4A), differentially expressed genes are enriched in muscle function-related pathways (muscle contraction, skeletal muscle contraction, creatine biosynthetic process), coagulation and platelet dysregulation pathways (hemostasis, blood coagulation, platelet activation, fibrin clot formation), and protein polymerization processes. KEGG analysis further demonstrates (Figure 4B) significant differential expression of genes in the FoxO signaling pathway, PI3K-Akt signaling pathway, and apoptosis pathway. GSEA analysis of the FoxO signaling and apoptosis pathways (Figures 4C,D) validates their regulatory roles in leukemia cell fate. These findings elucidate the multi-dimensional mechanistic actions of ATRA, involving coordinated disruption of muscle metabolism, dysregulated coagulation, and synergistic cell cycle-apoptosis modulation, providing molecular insights into its clinical antileukemic efficacy and potential thrombotic risks.

#### 3.4.3 Multi-pathway regulatory mechanisms of RIF in treating APL

**FIGURE 5:**
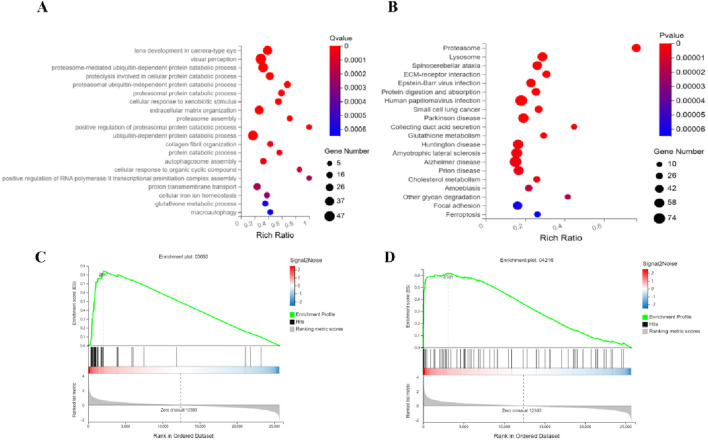
Synergistic targeting of proteasome and ferroptosis pathways underlies RIF’s anti-leukemic efficacy. Functional dissection identifies RIF-driven pathway perturbations: **(A)** GO analysis reveals coordinated dysregulation in ubiquitin-proteasome system (proteasome assembly, ubiquitin-dependent/independent degradation) coupled with autophagic flux and iron homeostasis; **(B)** KEGG analysis confirms proteasome activation (intracellular proteostasis hub) and ferroptosis-glutathione axis alterations; Gene Set Enrichment Analysis (GSEA) validates coherent modulation of **(C)** Proteasome and **(D)** Ferroptosis pathways. These findings highlight a dual-pathway mechanism where RIF suppresses leukemia progression by inhibiting proteasome hyperactivity and inducing iron-dependent redox stress.

Integrated analysis of the RIF-vs-Model group reveals key leukemia-associated functional perturbations. GO Biological Process enrichment (Figure 5A) highlights predominant dysregulation in ubiquitin-proteasome system (UPS)-related processes, including proteasome-mediated ubiquitin-dependent/independent protein degradation, proteasome assembly, and activation, together with autophagy-associated pathways (autophagosome assembly, macroautophagy) and iron ion homeostasis. KEGG pathway analysis (Figure 5B) confirms marked differential expression in the Proteasome pathway (a central hub for intracellular proteostasis linked to leukemia aggressiveness), alongside Ferroptosis and Glutathione metabolism, the latter two implicating redox imbalance and iron-dependent cell death. GSEA validation of Proteasome (Figure 5C) and Ferroptosis (Figure 5D) pathways underscores their coherent regulation. Collectively, RIF exerts anti-leukemic effects via dual-targeted suppression of proteasome-driven proteolytic hyperactivity and induction of ferroptosis-mediated metabolic disruption, offering mechanistic insights into its therapeutic efficacy against leukemia progression.

#### 3.4.4 Multi-pathway regulatory mechanisms of combination therapy in treating APL

**FIGURE 6:**
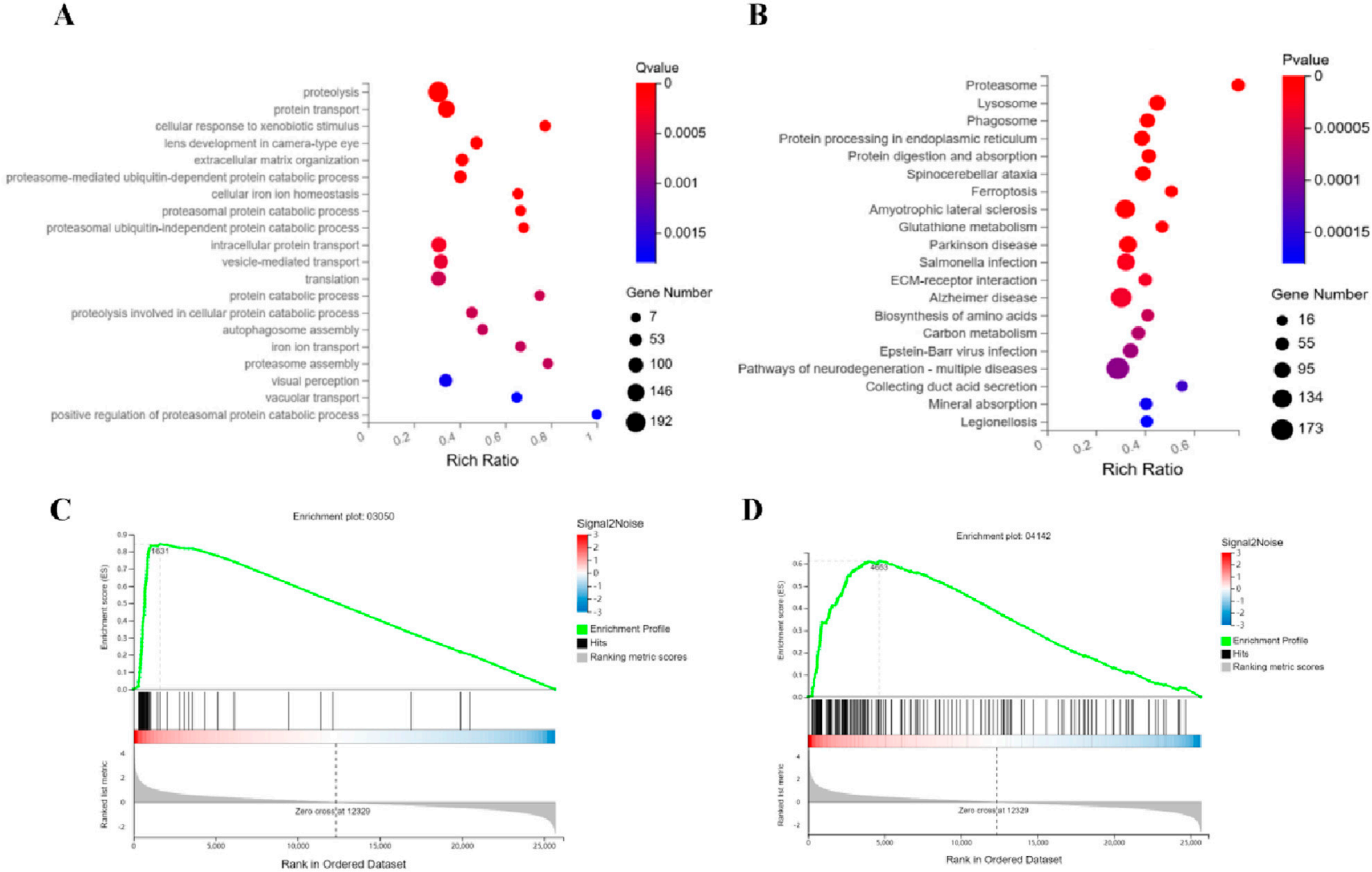
Mechanism of the combination therapy in synergistically suppressing APL through triple-axis targeting of the proteasome, ferroptosis, and lysosome pathways. **(A)** GO analysis reveals marked perturbations in ubiquitin-proteasome system (UPS) processes, including ubiquitin-dependent/independent proteolysis, proteasome assembly, and enhanced proteasomal degradation regulation, alongside disrupted iron ion homeostasis and autophagosome dynamics. **(B)** KEGG pathway enrichment indicates significant activation of the proteasome pathway (central to proteostasis), ferroptosis, glutathione metabolism (linked to oxidative stress), and concurrent enrichment of neurodegenerative disease pathways (e.g., Parkinson’s and Alzheimer’s). **(C,D)** GSEA validates robust modulation of the proteasome and lysosome pathways.

Integrated analysis of the Combination-vs-Model group reveals coordinated dysregulation of leukemia-associated molecular programs. GO Biological Process enrichment (Figure 6A) demonstrates pronounced perturbations in ubiquitin-proteasome system (UPS) processes, including proteasome-mediated ubiquitin-dependent/independent protein catabolism, proteasome assembly, and positive regulation of proteasomal degradation, accompanied by impaired iron ion homeostasis (cellular iron transport and balance) and autophagosome assembly dynamics. KEGG pathway mapping (Figure 6B) identifies significant alterations in the Proteasome pathway (a pivotal node for intracellular proteostasis), Ferroptosis, and Glutathione metabolism (key to oxidative stress regulation), with concurrent enrichment in neurodegenerative disease pathways (e.g., Parkinson disease, Alzheimer disease) suggesting potential cross-talk between leukemia progression and neuronal degeneration. GSEA further validates robust modulation of (Figure 6C) Proteasome and (Figure 6D) Lysosome pathways, the latter implicating autophagic-lysosomal interplay. Collectively, the combination therapy exerts anti-leukemic synergy through triple-pathway targeting: suppressing UPS-driven proteolytic hyperactivation, disrupting ferroptosis-associated redox equilibrium, and engaging lysosomal degradation machinery, thereby providing a multi-modal mechanistic framework for combating leukemia while revealing unexpected neurodegeneration-related signatures.

## 4 Discussion

All-trans retinoic acid (ATRA) plays a crucial role in the treatment of acute promyelocytic leukemia (APL) as a frontline therapy. ATRA, a derivative of vitamin A, specifically binds to the PML-RARα fusion protein, which is characteristic of APL (Tomita et al., 2013; Biswas et al., 2024). By releasing the inhibition of gene transcription imposed by the fusion protein, ATRA activates a series of genes associated with cell differentiation, inducing the maturation of leukemia cells and restoring their normal morphology and function (Li et al., 2018; Duan et al., 2024). This mechanism helps treat leukemia by promoting differentiation and inhibiting proliferation. Furthermore, ATRA regulates the expression of cell cycle proteins, causing cell cycle arrest at specific stages and suppressing leukemia cell proliferation, research have found that during the development of BPDCN leukemia, MYB switches from a regulator of DC lineage genes to aberrantly regulating G2/M cell cycle control genes. While ATRA may lead to the loss of MYB protein and cell death in BPDCN (Booth et al., 2024; Rehman et al., 2024). Additionally, ATRA can modulate immune function, decrease the Th17 cell immune response, enhancing the body’s ability to monitor and eliminate tumor cells (Padua et al., 2024; Wørzner et al., 2024). Clinical studies have shown that ATRA monotherapy achieves a complete remission rate of 80%–90% in APL patients, greatly improving their prognosis. However, resistance to ATRA remains a challenge when used alone. A 13 - year clinical study on the first - line treatment of APL patients with single - agent liposomal - encapsulated ATRA found that the overall complete remission (CR) rate was 79%. The CR rates of low - risk patients (with an initial white blood cell count <10,000) and high - risk patients (with a white blood cell count ≥10,000) were 92% and 38%, respectively (Jain et al., 2013).

RIF (Realgar-Indigo Naturalis formula), a traditional Chinese medicine compound, works through multiple targets and pathways to enhance the therapeutic effects on leukemia and improve chemoresistance. It has demonstrated significant benefits in improving clinical symptoms and the quality of life for patients (Lou et al., 2021; Fang et al., 2024; Chen and Ding, 2024). In this study, a zebrafish leukemia model was used to systematically investigate the anti-tumor and liver injury improvement effects of RIF. Transcriptomic analysis was conducted to further elucidate its potential mechanisms of action. The results showed that RIF significantly inhibited tumor growth by reducing the fluorescence intensity of HL-60 cells and alleviated liver injury induced by HL-60 cells, evidenced by the reduced fatty vacuolar degeneration in liver tissue. Additionally, transcriptomic analysis revealed that ATRA and RIF exert their effects through distinct signaling pathways and produce synergistic effects when combined.

Transcriptomic analysis indicated that ATRA primarily impacts the FoxO signaling pathway, PI3K-Akt signaling pathway, apoptosis, and complement and coagulation cascades. The FoxO pathway is critical for regulation, apoptosis, and DNA damage repair. ATRA has been shown to activate this pathway to promote differentiation and inhibit proliferation in leukemia cells (Zhang et al., 2011; Guan et al., 2025; Sun et al., 2024). The PI3K-Akt pathway, which is involved in cell survival and proliferation, is regulated by ATRA to suppress leukemia cell growth by lowering Akt phosphorylation (Swords et al., 2015; Zhang M. et al., 2025). The activation of complement and coagulation cascades may play a role in ATRA-induced regulation of the leukemia microenvironment, further promoting tumor cell clearance (Le et al., 2025; Tang et al., 2024). In contrast, RIF mainly influences the ubiquitin-proteasome system, ferroptosis, and glutathione metabolism. The ubiquitin-proteasome system is vital for maintaining cellular homeostasis and protein degradation (Wei et al., 2024). RIF likely enhances the degradation of abnormal proteins in leukemia cells, thus inhibiting their proliferation. Ferroptosis, a non-apoptotic form of cell death, is closely linked to dysregulated iron metabolism and reactive oxygen species (ROS) accumulation (Yu et al., 2024; Akiyama et al., 2024). This study suggests that RIF may promote ferroptosis, increasing leukemia cell sensitivity and inhibiting their growth. Furthermore, RIF significantly affects glutathione metabolism, which could reduce antioxidant capacity and further promote ferroptosis.

When ATRA and RIF are used in combination, they also significantly affect autophagosome and lysosome pathways. Autophagy is an essential process for maintaining cellular homeostasis and can play a role in both tumor cell survival and death (Zhang N. et al., 2025; Zhu et al., 2025). Lysosomes are crucial in autophagic flow and intracellular degradation (Kroemer and Jäättelä, 2005). The combination of ATRA and RIF may enhance autophagy and lysosomal-mediated degradation, accelerating leukemia cell clearance. Additionally, the potential interaction between autophagy and ferroptosis should not be overlooked, as research suggests that autophagy regulates ferroptosis, the combined treatment in this study may enhance the anti-leukemia effects through this mechanism (Liu et al., 2020).

Regarding liver injury, the model control group exhibited prominent fatty vacuolar degeneration in the liver, while RIF treatment significantly alleviated liver damage. Transcriptomic analysis suggested that RIF might regulate the ubiquitin-proteasome system and glutathione metabolism to reduce oxidative stress and improve pathological changes in liver tissue. Ferroptosis has gained attention in liver injury research, and RIF may modulate ferroptosis to reduce liver cell damage, thus exerting a protective effect.

Our study utilized a zebrafish xenograft model, which, as a robust platform, offers significant advantages for rapid drug screening and initial mechanistic investigations, particularly due to the optical transparency of larvae and the high conservation of oncogenic pathways. However, we fully acknowledge the inherent limitations of this model in completely recapitulating the intricacies of human leukemia. Specifically, the larval zebrafish model used in our experiments (up to 5 dpf) possesses an innate but immature adaptive immune system. This means that complex interactions between the therapeutic agents and the host immune microenvironment—a critical factor in human leukemia progression and treatment response—are not fully captured. Furthermore, the short experimental window in this acute model precludes the assessment of long-term disease progression, the development of acquired drug resistance, and chronic toxicity, all of which are vital considerations in the clinical management of APL.

Despite these limitations, our findings provide a valuable preclinical foundation by identifying key conserved signaling pathways (e.g., ubiquitin-proteasome, ferroptosis) fundamental to cell biology. To bridge the gap from this high-throughput model to clinical relevance, the logical next step is to validate our key findings in mammalian models, such as murine APL xenografts. Such models can better mimic the human tumor microenvironment and allow for longitudinal studies of efficacy and resistance. Investigating the effects of RIF on immune cell populations (e.g., T-cells, macrophages) and their interplay with leukemia cells within these mammalian systems would be a particularly insightful future direction. Ultimately, corroborating our findings in more complex preclinical settings is essential to pave the way for potential clinical trials.

In summary, this study provides a comprehensive exploration of RIF’s role in leukemia treatment, from animal models to molecular levels and signaling pathways. RIF exerts dual effects by modulating the ubiquitin-proteasome system, ferroptosis, and glutathione metabolism, providing anti-leukemia and liver injury-improving benefits. When combined with ATRA, RIF further enhances autophagy and lysosomal-mediated degradation. These findings not only offer new theoretical support for the application of RIF in leukemia treatment but also provide new insights for future combination therapy strategies. While our comprehensive transcriptomic analysis provides a robust map of the molecular pathways affected by RIF and ATRA, we acknowledge a key limitation is the absence of direct experimental validation of these findings within this manuscript. Future studies should prioritize validating the expression changes and functional relevance of key proteins within the ubiquitin-proteasome system, ferroptosis pathways, and the autophagy-lysosome axis using established orthogonal methods such as qRT-PCR to confirm mRNA levels and, critically, Western blotting to verify changes at the protein level (e.g., key proteasome subunits, regulators of ferroptosis such as GPX4, and autophagy markers like the LC3-II/LC3-I ratio), thus promoting its clinical translation in leukemia treatment.

## 5 Conclusion

This study demonstrates that the Realgar-Indigo Naturalis formula (RIF) exerts potent anti-leukemic effects in a zebrafish HL-60 tumor model, reducing tumor burden and alleviating HL-60-induced liver injury. Transcriptomic profiling revealed that RIF targets the ubiquitin-proteasome system, ferroptosis, and glutathione metabolism, while ATRA primarily modulates FoxO and PI3K-Akt pathways. Their combination further engages autophagosome-lysosome crosstalk, unveiling a synergistic multi-pathway mechanism. RIF’s multimodal action—suppressing proteolytic hyperactivation, disrupting redox imbalance, and activating lysosomal degradation—positions it as a promising therapeutic candidate with potential clinical advantages over single-target therapies. These findings bridge traditional medicine and molecular oncology, highlighting RIF’s value in leukemia treatment and warranting further translational validation.

## Data Availability

The original contributions presented in the study are included in the article/supplementary material, further inquiries can be directed to the corresponding author.
